# Supplementary material for: Analysis of false-negative rapid diagnostic tests for symptomatic malaria in the Democratic Republic of the Congo
Source: Sci Rep. 2021 Mar 22;11:6495. doi: 10.1038/s41598-021-85913-z (PMC7985209; doi:10.1038/s41598-021-85913-z)
Supplement: Supplementary file 2 — Supplementary Information 2. [file 41598_2021_85913_MOESM2_ESM.docx]

**SUPPLEMENT TO:**

**Analysis of false-negative rapid diagnostic tests for symptomatic malaria in the Democratic Republic of the Congo**

**Authors:** Jonathan B. Parr,^1^*^#^ Eddy Kieto,^2^* Fernandine Phanzu,^2^ Paul Mansiangi,^3^ Kashamuka Mwandagalirwa,^3^ Nono Mvuama,^3^ Ange Landela,^4^ Joseph Atibu,^3^ Solange Umesumbu Efundu,^5^ Jean W. Olenga,^2^ Kyaw Lay Thwai,^1^ Camille E. Morgan,^6^ Madeline Denton,^1^ Alison Poffley,^6^ Jonathan J. Juliano,^1,6^ Pomie Mungala,^2^ Joris L. Likwela,^2^ Eric M. Sompwe,^5^ Eric Rogier,^7^ Antoinette K. Tshefu,^3^ Adrien N’Siala,^2^ Albert Kalonji^2^

* Co-first authors

**Affiliations:**

1. Institute for Global Health and Infectious Diseases, University of North Carolina, Chapel Hill, NC 27599, USA.
2. SANRU Asbl (Sante Rurale/Global Fund), Kinshasa, Democratic Republic of the Congo.
3. University of Kinshasa School of Public Health, Kinshasa, Democratic Republic of Congo.
4. Institut National pour La Recherche Biomedicale, Kinshasa, Democratic Republic of the Congo.
5. Programme National de la Lutte contre le Paludisme, Kinshasa, Democratic Republic of Congo.
6. Department of Epidemiology, Gillings School of Global Public Health, University of North Carolina, Chapel Hill, NC 27599, USA.
7. Malaria Branch, Division of Parasitic Diseases and Malaria, Centers for Disease Control and Prevention, Atlanta, GA 30033, USA.

**Running head:** Causes of false-negative malaria RDTs in the DRC

**Key words:** rapid diagnostic tests, histidine-rich protein 2, pfhrp3, hrp2, hrp3, RDT, deletion, malaria, *Plasmodium falciparum, Plasmodium ovale, Plasmodium malariae, Plasmodium vivax,* Congo

**^#^ Corresponding author:** Jonathan B. Parr, MD, MPH, Division of Infectious Diseases, University of North Carolina, 130 Mason Farm Rd., Chapel Hill, NC 27599; phone 1-919-445-1132, email jonathan_parr@med.unc.edu

**Supplementary Table 1. Characteristics of study subjects by health zone.**

| Province | **Overall** |  | **Bas-Uele** |  |  | **Kinshasa** |  |  | **Sud-Kivu** |  |
| --- | --- | --- | --- | --- | --- | --- | --- | --- | --- | --- |
| Health zone |  |  | Buta | Ganga |  | Limete | Nsele |  | Idjwi | Kadutu |
| n | 3627 |  | 598 | 605 |  | 629 | 619 |  | 587 | 589 |
| Age, median years (IQR) | 18 (4, 32) |  | 15 (3, 28) | 19 (3, 32) |  | 21 (3.5, 30.5) | 21 (4, 35) |  | 16 (4, 36) | 15 (5, 34) |
| Age strata, n (%) |  |  |  |  |  |  |  |  |  |  |
| <5 years | 1025 (28.7) |  | 177 (30.7) | 193 (32.1) |  | 188 (29.9) | 147 (23.7) |  | 179 (30.5) | 141 (25.2) |
| 5-14 years | 579 (16.2) |  | 107 (18.5) | 63 (10.5) |  | 115 (18.3) | 156 (25.2) |  | 51 (8.7) | 87 (15.6) |
| 15-24 years | 639 (17.9) |  | 116 (20.1) | 116 (19.3) |  | 95 (15.1) | 86 (13.9) |  | 133 (22.7) | 93 (16.6) |
| 25-34 years | 513 (14.4) |  | 79 (13.7) | 91 (15.1) |  | 64 (10.2) | 80 (12.9) |  | 107 (18.2) | 92 (16.5) |
| 35-44 years | 344 (9.6) |  | 34 (5.9) | 67 (11.1) |  | 64 (10.2) | 67 (10.8) |  | 50 (8.5) | 62 (11.1) |
| 45-54 years | 249 (7.0) |  | 37 (6.4) | 36 (6.0) |  | 66 (10.5) | 42 (6.8) |  | 31 (5.3) | 37 (6.6) |
| 55 years and older | 223 (6.2) |  | 27 (4.7) | 35 (5.8) |  | 37 (5.9) | 41 (6.6) |  | 36 (6.1) | 47 (8.4) |
| Female gender, n (%) | 2130 (58.7) |  | 403 (67.4) | 377 (62.3) |  | 302 (48.0) | 344 (55.6) |  | 342 (58.3) | 362 (61.5) |
| Pregnant, n (%) | 350 (16.4) |  | 81 (20.1) | 24 (6.4) |  | 4 (1.3) | 22 (6.4) |  | 119 (34.8) | 100 (27.6) |
| Slept under bednet the night before, n (%) | 2238 (79.5) |  | 396 (93.8) | 190 (54.3) |  | 480 (85.6) | 422 (76.2) |  | 355 (85.7) | 395 (77.0) |
| Diagnosed with malaria in the last six months, n (%) | 1556 (43.1) |  | 390 (65.2) | 368 (60.8) |  | 173 (28.3) | 289 (46.8) |  | 196 (33.4) | 140 (23.8) |
| Microscopy-positive, n (%) | 1397 (38.7) |  | 189 (31.7) | 311 (51.6) |  | 246 (39.5) | 287 (46.6) |  | 298 (50.8) | 66 (11.2) |
| RDT-positive, n (%) | 1545 (42.6) |  | 334 (55.9) | 424 (70.1) |  | 99 (15.7) | 281 (45.4) |  | 342 (58.3) | 65 (11.0) |
| RDT-negative, microscopy-positive, n (%) | 426 (11.8) |  | 14 (2.3) | 37 (6.1) |  | 178 (28.5) | 89 (14.4) |  | 69 (11.8) | 39 (6.6) |

**Supplementary Table 2. Characteristics of pfhrp2/3-negative subjects by initial PCR genotyping.** These subjects were ultimately not classified as pfhrp2/3-deleted based on the results of whole-genome sequencing (WGS) and antigenemia assessment. All eight had evidence of intact genes by WGS and HRP2 antigenemia by Luminex. Abbreviations: NA, not applicable; micro, microscopy.

| **Sample ID** | ***Pfhrp2/3* PCR genotype** | **Province** | **Health zone** | **Health area** | **Age, years** | **Gender** | **Pregnancy status** | **Micro result** | **RDT result** | ***Pfldh* qPCR, parasites/µL** |
| --- | --- | --- | --- | --- | --- | --- | --- | --- | --- | --- |
| SANHRP_01 | *pfhrp2-/3-* | Kinshasa | Nsele | Pecheur | 43 | Female | Pregnant | + | - | 144 |
| SANHRP_02 | *pfhrp2-/3-* | Kinshasa | Nsele | Mikonga | 12 | Male | NA | + | - | 3,018 |
| SANHRP_03 | *pfhrp2-/3-* | Sud-Kivu | Kadutu | Ciriri | 21 | Female | Not pregnant or unknown | + | - | 1,705 |
| SANHRP_04 | *pfhrp2-/3-* | Sud-Kivu | Kadutu | Funu | 45 | Female | Pregnant | + | + | 84 |
| SANHRP_05 | *pfhrp2+/3-* | Sud-Kivu | Idjwi | Mpene | 23 | Female | Not pregnant or unknown | + | + | 8,826 |
| SANHRP_06 | *pfhrp2-/3-* | Sud-Kivu | Idjwi | Mpene | 25 | Female | Not pregnant or unknown | + | - | 2,842 |
| SANHRP_07 | *pfhrp2-/3+* | Sud-Kivu | Idjwi | Mafula | 3 | Female | Not pregnant or unknown | + | + | 102,700 |
| SANHRP_08 | *pfhrp2-/3+* | Sud-Kivu | Idjwi | Mafula | 2 | Female | Not pregnant or unknown | + | - | 3,154 |

**Supplementary Table 3. F_ws_ values suggest monoclonal infection in a minority (n=3/8) of samples subjected to whole-genome sequencing.** Samples with F_ws_ values >0.95 were considered monoclonal infections.

| **Sample ID** | **Fws** | **Standard error** | **Sites analyzed, n** | **COI** |  | **BioProject ID** | **BioSample accession** |
| --- | --- | --- | --- | --- | --- | --- | --- |
| SANHRP_01 | 0.887 | 0.017 | 40,780 | >1 |  | PRJNA675671 | SAMN16711875 |
| SANHRP_02 | 0.972 | 0.042 | 40,818 | 1 |  | PRJNA675671 | SAMN16711876 |
| SANHRP_03 | 0.958 | 0.035 | 40,735 | 1 |  | PRJNA675671 | SAMN16711877 |
| SANHRP_04 | 0.946 | 0.026 | 40,774 | >1 |  | PRJNA675671 | SAMN16711878 |
| SANHRP_05 | 0.581 | 0.005 | 40,771 | >1 |  | PRJNA675671 | SAMN16711879 |
| SANHRP_06 | 0.630 | 0.006 | 40,870 | >1 |  | PRJNA675671 | SAMN16711880 |
| SANHRP_07 | 0.960 | 0.032 | 40,692 | 1 |  | PRJNA675671 | SAMN16711881 |
| SANHRP_08 | 0.769 | 0.011 | 40,885 | >1 |  | PRJNA675671 | SAMN16711882 |

**Supplementary Table 4. Comparison of malaria diagnostic test results:** HRP2-based RDT, *P. falciparum* 18S rRNA real-time PCR, and microscopy (micro) comparisons, counts (percent). A) RDT versus PCR. B) RDT versus microscopy. C) Microscopy versus PCR. D) RDT and PCR profiles by province. Abbreviations: HRP2, histidine-rich protein 2; RDT, rapid diagnostic test; rRNA, ribosomal RNA; PCR, polymerase chain reaction.

| **A.** |  |  |  | **PCR+** | **PCR-** | |  | |  | |  |
| --- | --- | --- | --- | --- | --- | --- | --- | --- | --- | --- | --- |
|  |  | **RDT+** |  | 404 (40) | 36 (4) | |  | |  | |  |
|  |  | **RDT-** |  | 134 (13) | 426 (43) | |  | |  | |  |
|  |  |  |  |  |  | |  | |  | |  |
| **B.** |  |  |  | **Micro+** | **Micro-** | |  | |  | |  |
|  |  | **RDT+** |  | 267 (27) | 173 (17) | |  | |  | |  |
|  |  | **RDT-** |  | 108 (11) | 452 (45) | |  | |  | |  |
|  |  |  |  |  |  | |  | |  | |  |
| **C.** |  |  |  | **PCR+** | **PCR-** | |  | |  | |  |
|  |  | **Micro+** |  | 287 (29) | 88 (9) | |  | |  | |  |
|  |  | **Micro-** |  | 251 (25) | 374 (37) | |  | |  | |  |
|  |  |  |  |  |  | |  | |  | |  |
| **D.** |  |  |  | **RDT-/PCR-** | | **RDT-/PCR+** | | **RDT+/PCR+** | | **RDT+/PCR-** | |
|  | **Province** | Kinshasa |  | 234 (66) | | 17 (5) | | 87 (25) | | 15 (4) | |
|  |  | Sud-Kivu |  | 142 (45) | | 54 (17) | | 112 (35) | | 11 (3) | |
|  |  | Bas-Uele |  | 50 (15) | | 63 (19) | | 205 (62) | | 10 (3) | |

**Supplementary Figure 1. Distribution of bead-based immunoassay signals for a panel of 92 blood samples collected from donors without malaria.** Histograms shown for log-transformed median fluorescence intensity minus background (MFI-bg) assay signal for A) HRP2, B) pLDH, and C) pAldolase, with hashed vertical line indicating the positivity threshold signal for each.

**
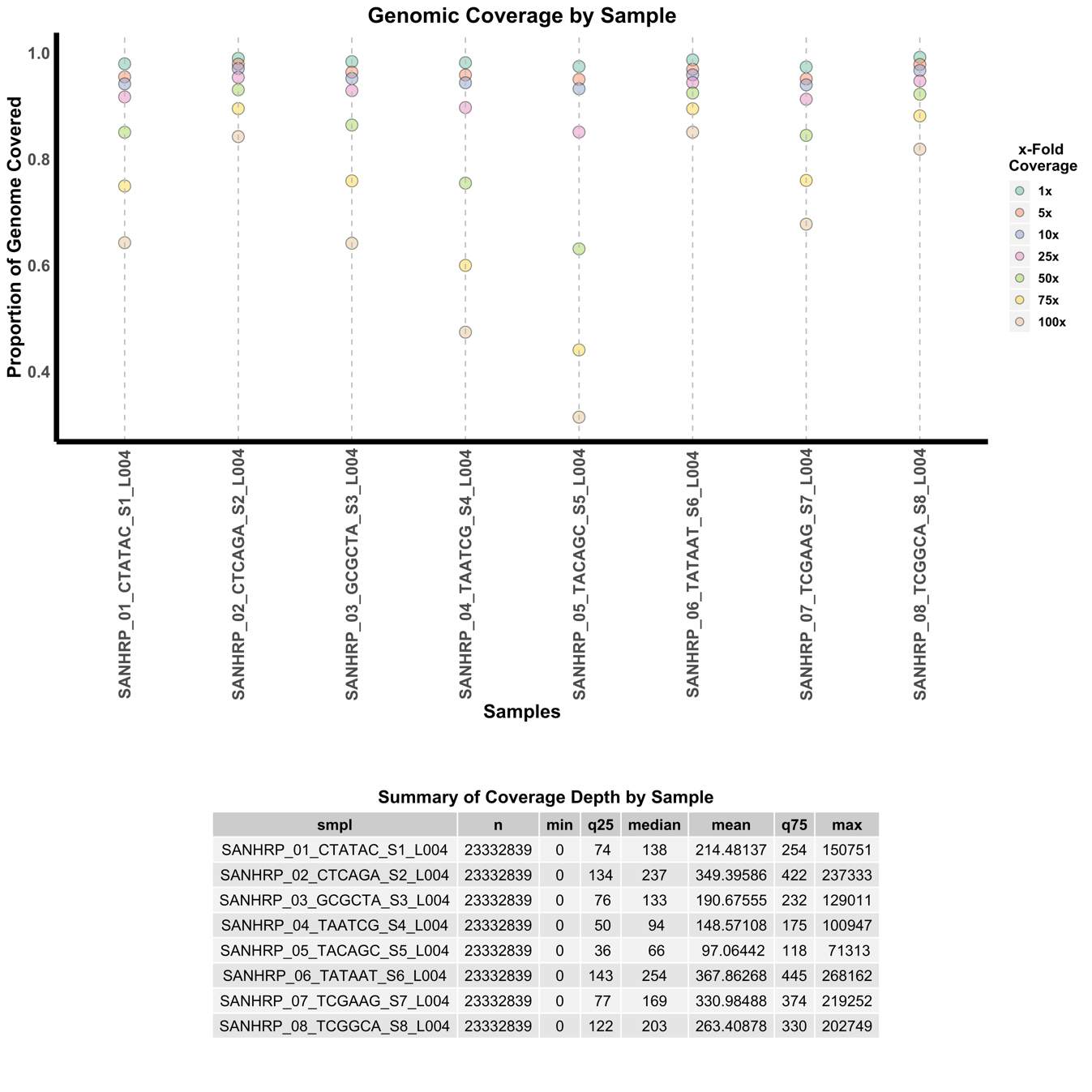
**

**Supplementary Figure 2. Genomic coverage statistics by sample.** During initial PCR genotyping, samples SANHRP_01, 02, 03, 04, and 06 were *pfhrp2-/3-;* 05 was *pfhrp2+/3-*; and 07 and 08 were *pfhrp2-/3+* (see Supplementary Table 2)*.* However, WGS and antigen testing confirmed the presence of intact genes. Coverage included regions with very high coverage (“jackpotting”), a finding not unexpected after selective whole-genome amplification (sWGA).

**Supplementary Figure 3. Log_10_-transformed Luminex antigen values by RDT result.** Thresholds for positivity (see Supplementary Figure 1) are displayed as dashed horizontal lines. One pLDH value with background-subtracted MFI <0 is not depicted. Abbreviations: MFI, mean fluorescence intensity.

32% PCR+

55% PCR+

85% PCR+

**Supplementary Figure 4**. **Malaria prevalence was high (56.8% overall) and non-falciparum co-infection with *P. falciparum* common (13.2% overall) among symptomatic subjects.** *Plasmodium* PCR prevalence and species-specific real-time PCR results for *P. falciparum* (Pf)*, P. ovale* (Po)*, P. malariae* (Pm)*,* and *P. vivax* (Pv) among 1,000 samples tested, with counts reported by province. Province-level prevalence of pan-*Plasmodium* PCR-positivity is displayed in white font.


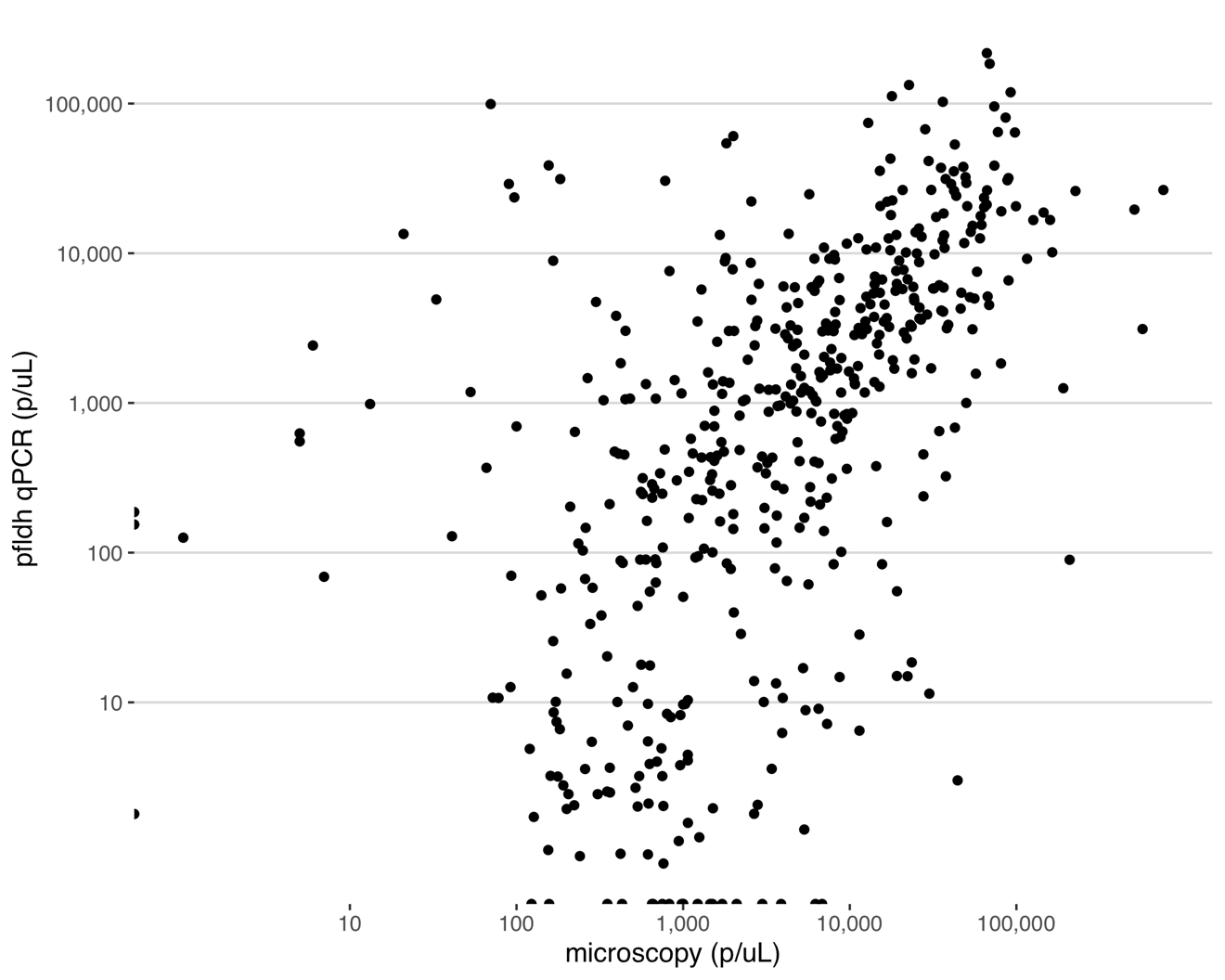


**Supplementary Figure 5. Parasite densities determined by *pfldh* qPCR and microscopy were similar**, with moderate correlation (Spearman correlation coefficient = 0.63, p <0.001). Abbreviations: p/uL, parasites/microliter.

**Supplementary File. PCR primers, probes, and reaction conditions.** See separate Excel file.
